# Valganciclovir for Cytomegalovirus Prevention in Solid Organ Transplant Patients: An Evidence-Based Reassessment of Safety and Efficacy

**DOI:** 10.1371/journal.pone.0005512

**Published:** 2009-05-13

**Authors:** Andre C. Kalil, Alison G. Freifeld, Elizabeth R. Lyden, Julie A. Stoner

**Affiliations:** 1 Infectious Diseases Division, Internal Medicine Department, University of Nebraska Medical Center, Omaha, Nebraska, United States of America; 2 Department of Biostatistics, College of Public Health, University of Nebraska, Omaha, Nebraska, United States of America; 3 Department of Biostatistics and Epidemiology, College of Public Health, University of Oklahoma Health Sciences Center, Oklahoma City, Oklahoma, United States of America; INSERM, France

## Abstract

**Background:**

Several anti-viral drugs have demonstrated efficacy in preventing Cytomegalovirus (CMV) infections in solid organ transplant (SOT) patients. The recently approved valganciclovir is the most commonly used and most expensive drug for CMV prevention. The safety and efficacy data have been drawn from a single trial. We hypothesized that valganciclovir may not be as safe as nor more effective than other therapies for CMV prevention.

**Methods:**

All experimental and analytical studies that compared valganciclovir with other therapies for prevention of CMV infection after SOT were selected. Based on meta-analytic and multivariate regression methodologies we critically analyzed all available evidence.

**Findings:**

Nine studies were included (N = 1,831). In trials comparing valganciclovir with ganciclovir, the risk for CMV disease is 0.98 (95% Confidence Interval (95%CI) 0.67 to 1.43; P = 0.92; I^2^ = 0%). Valganciclovir was significantly associated with the risk of absolute neutropenia (<1,500/mm^3^) compared with all therapies (Odds Ratio (OR) 3.63 95%CI 1.75 to 7.53; P = 0.001; I^2^ = 0%); with ganciclovir only (OR 2.88, 95%CI 1.27 to 6.53; P = 0.01; I^2^ = 0%); or with non-ganciclovir therapies (OR 8.30, 95%CI 1.51 to 45.58; P = 0.01; I^2^ = 10%). For a neutropenia cut-off of <1,000/mm^3^, the risk remained elevated (OR 1.97, 95%CI 1.03 to 3.67; P = 0.04; I^2^ = 0%). For every 24 patients who receive valganciclovir prophylaxis, one more will develop neutropenia compared to other therapies. The risk of late-onset CMV disease with valganciclovir was similar to ganciclovir and higher than those with non-ganciclovir therapies (OR 8.95, 95%CI 1.07 to 74.83; P = 0.04; I^2^ = 0%]. One more patient will develop late-onset CMV disease for every 25 who receive valganciclovir compared to treatment with non-ganciclovir therapies. The risk of CMV tissue-invasive disease in liver recipients receiving valganciclovir was 4.5 times the risk seen with ganciclovir [95%CI 1.00 to 20.14] (p = 0.04). All results remained consistent across different study designs, valganciclovir doses, and CMV serostatus.

**Conclusions:**

Valganciclovir shows no superior efficacy and significantly higher risk of absolute neutropenia, CMV late-onset disease, and CMV tissue-invasive disease compared to other standard therapies. Due to the availability of efficacious, safer, and lower cost drugs (high-dose acyclovir, valacyclovir, ganciclovir), our results do not favor the use of valganciclovir as a first-line agent for CMV preemptive or universal prophylaxis in SOT patients.

## Introduction

Cytomegalovirus (CMV) is the most frequent opportunistic infection in solid organ transplant (SOT) patients, causing either CMV syndrome (fever, malaise and cytopenia) or CMV disease usually in the first year post-transplant [1], [2]. Several approaches have evolved to prevent this infection, including universal prophylaxis with anti-viral agents (i.e. acyclovir, valacyclovir, ganciclovir, valganciclovir), and pre-emptive strategy with ganciclovir or valganciclovir. Efficacy superiority has not been demonstrated for either a specific strategy or anti-viral drug in numerous clinical trials and meta-analyses [2]–[4]. Nonetheless, valganciclovir is the most widely employed drug for pre-emptive and universal prophylaxis, used in approximately two-thirds of all SOT patients [5], [6]. The reasons for this popularity are multifactorial, including the convenience of once daily dosing, limitations on the production of oral ganciclovir, and influential marketing strategies by the manufacturer. Despite its commercial success, we hypothesize that valganciclovir may be less safe and not more effective than its substantially less expensive alternatives, oral ganciclovir, oral acyclovir or valacyclovir for the prevention of CMV.

Valganciclovir (L-valyl ester prodrug of ganciclovir with higher bioavailability than oral ganciclovir) received FDA approval in September of 2003 for the prevention of CMV infection in high-risk (defined as CMV seronegative recipients of organs from CMV seropositive donors) kidney, kidney-pancreas and heart transplant recipients based on a non-inferiority trial comparing this drug with oral ganciclovir. The trial by Paya et al [7] showed that valganciclovir was not inferior to ganciclovir for transplant recipients at high risk for cytomegalovirus. A notable exception was observed in liver recipients in whom a significantly higher rate of CMV invasive-tissue disease occurred in those receiving valganciclovir compared with the ganciclovir recipients; accordingly, the FDA did not approve valganciclovir for prophylaxis following liver transplantation [8]. Furthermore, the same trial suggested that neutropenia may be an important adverse effect of valganciclovir prophylaxis, affecting 8% of those taking the drug [7].

Since the original trial [7] was published, many subsequent clinical studies using valganciclovir for either pre-emptive or universal prophylaxis in solid organ transplant recipients have been published [9]–[24]. Recognizing that the single, non-inferiority original trial [7] cannot address all clinically relevant issues, we undertook a meta-analysis of all available data from both this pivotal trial and from more recently published studies to extend our knowledge about the safety and efficacy of valganciclovir prophylaxis in the setting of solid organ transplantation.

The efficacy aim of our study is to determine the reduction in CMV disease and the safety aim is to determine the risks of neutropenia, opportunistic infections, late-onset CMV disease, and death among patients receiving valganciclovir versus other preventive therapies (i.e. ganciclovir, valacyclovir, and high-dose acyclovir), or approaches (i.e. prophylaxis and preemptive).

## Materials and Methods

### Literature Search

A systematic literature search was performed without language restrictions from inception to May 2008 in the following databases: Medline, Embase, and Cochrane Library. In addition, we searched abstracts published in the same time period from the following meetings: Infectious Diseases Society of America, American Transplantation Congress, and the Interscience Conference on Antimicrobial Agents and Chemotherapy. Relevant internet sites such as the Food and Drug Administration reports [8] and trial results repositories (www.clinicalstudyresults.org and www.clinicaltrialresults.org) were also searched. The keywords used were valganciclovir, valcyte, cytomegalovirus, prevention, prophylaxis, preemptive, organ, lung, pulmonary, kidney, renal, liver, hepatic, heart, cardiac, pancreas, transplant, and transplantation.

### Study Selection

Inclusion Criteria: All experimental (randomized), and analytical (cohort and case-control) studies which primarily aimed to compare valganciclovir with other therapies or approaches for prevention of CMV disease after SOT were selected for both safety and efficacy analyses.

Exclusion Criteria: Non-comparative observational studies were not included in the meta-analysis, but were discussed as descriptive data.

### Data Extraction

A standardized form was used to abstract and collect the following variables: authors; publication year; study design; type of allograft; gender; mean age; sample size; CMV serostatus; induction therapy; maintenance immunosuppressive therapy; valganciclovir regimen; comparator regimen; length of CMV prophylaxis; length of follow up; white blood cell count; neutrophil count; CMV viremia; CMV syndrome; CMV disease; opportunistic infections; and survival. Any disagreement was resolved by further review of the study and consensus among two authors (A.C.K and A.G.F).

### Safety and Efficacy Definitions

Neutropenia: Absolute neutrophil count (ANC) less than 1,000 to 1,500 cells/mm^3^, which is considered grade 2 toxicity on the National Cancer Institute toxicity criteria [25].

CMV Disease: The presence of CMV syndrome (viral detection with fever, malaise, or cytopenia) and/or end-organ disease involvement by cytomegalovirus [26].

CMV Tissue-Invasive Disease: The presence of end-organ disease involvement by cytomegalovirus [26].

Late-Onset CMV Disease: The occurrence of CMV disease after the completion of universal prophylaxis.

Deaths: All-cause mortality.

### Statistical Analysis

**Figure 1:**
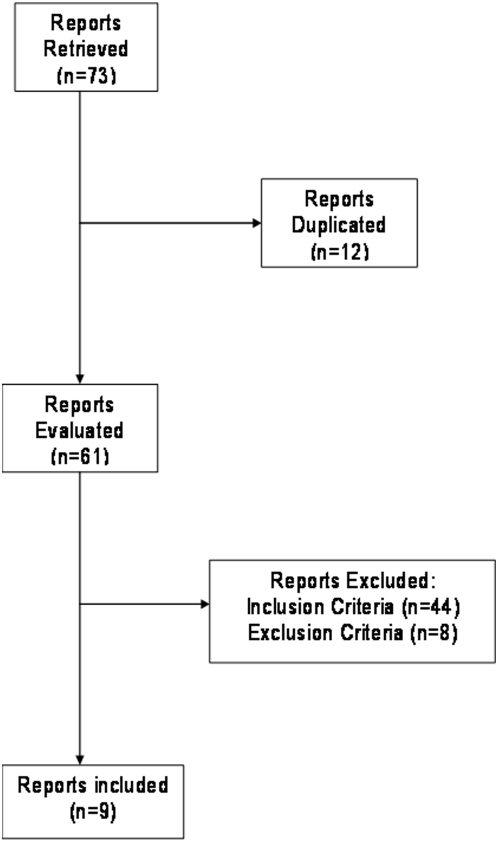
Quorom Trial Flow.

The data was pooled by using the Mantel-Haenszel fixed-effects model [27] and the DerSimonian and Laird [28] random-effects model. The Q statistic method was used to assess statistical heterogeneity and the I-squared method to assess the magnitude of variation secondary to heterogeneity [29]. All results were reported with the fixed-effects model, except when significant heterogeneity (p<0.1 or I^2^>50%) was detected. For studies with no event of interest in a treatment group, 1.0 was added to all cells. The software used was Comprehensive Meta-Analysis version 2.0 (Biostat, Englewood, New Jersey). We performed multivariate logistic regression analyses using the data from the FDA [8] and from the published data from this trial [7], [30], on overall CMV disease and tissue invasive CMV at 6 and 12 months. The analysis evaluated only liver and kidney transplants, since these were groups with the largest sample sizes, thus resulting in the most stable parameter estimates. Censoring due to death could not be performed because we did not have patient-level mortality information on when or for which groups deaths occurred before 6 months. In accordance with the Consolidated Standards of Reporting Trials guidelines [31], we relied on interactions between treatment and subgroups with the aim to avoid misinterpretation and to better understand the trial results for subgroups of patients. Because of the known conservative nature of this test, an interaction test with a p value less than 0.1 is considered statistically significant. The software used was SAS version 9.1.3 (SAS Institute, Cary, North Carolina). The number needed to treat and the number needed to harm were based on the odds ratios and control arm events rate of the included trials [32]. Since the event rates are low across the treatment groups and subset analyses, the odds ratio (OR) is used to estimate the relative risk (RR) and the treatment group effect is described in terms of risk instead of odds. The Jadad score was used to evaluate the quality of randomized studies (table 1), the QUOROM criteria for the search methodology (Figure 1), and the MOOSE checklist (Table S1) was completed to evaluate the quality of our study report. The cohort and case-control studies were not scored because there are no validated scoring systems for these study designs. Funnel plot, Egger regression and the Begg and Mazumdar methods were used to evaluate publication bias [32]–[34]. Statistical power calculations were performed based on the comparison of two independent proportions using the software PASS version 2005 (Number Crunching Statistical Systems, Kaysville, Utah).

**Table 1:** Characteristics of Studies.

AUTHOR	YEAR	DESIGN	ALLOGRAFT	SAMPLE SIZE	MEAN AGE (years)	GENDER Male/Female (%)	CMV D+/R− (%)	INDUCTION THERAPY (%)	FK (%)	CsA (%)	MMF (%)	AZT (%)	Prednisone (%)	VALGAN DAILY DOSE (MG)	CONTROL	PROPHYLAXIS DURATION (Days)	FOLLOW UP (Months)
																	Jadad Score
Paya C et al [7]	2004	Randomized	Kidney; Kidney-pancreas; Liver;Heart	364	45	74/26	100	n/a	n/a	n/a	n/a	n/a	n/a	900	Ganciclovir PO (3 g/day)	100	6 and 12
																	Jadad = 3
Khoury J et al [9]	2006	Randomized	Kidney	98	49	49/51	30	97	98	2	68	0	100	900	Preemptive	100	12
																	Jadad = 2
Said T et al [10]	2007	Randomized	Kidney	110	40	61/39	n/a	100	45	35	100	0	100	900	Ganciclovir IV (5 mg/kg/day)	14–90	6
																	Jadad = 2
Humar A et al [11]	2005	Cohort	Lung	80	49	66/44	25	29	0	90	54	36	100	900	Ganciclovir PO (3 g/day)	90	6 and 12
																	Jadad = NA
Zamora M et al [12]	2004	Cohort	Lung	230	54	55/45	21	0	0	100	0	100	100	Ganciclovir IV (30 days)+CMVIG (3 doses)+Valgan 900 mg (N = 11); 450 mg (N = 79)	Ganciclovir IV (30 days)+CMVIG (3 doses)+Acyclovir PO 800 TID	180–365	12
																	Jadad = NA
Weng F et al [13]	2007	Cohort	Kidney and Kidney-pancreas	500	46	58/62	21	55	31	61	84	0	77	450	Ganciclovir PO (3 g/day)	90	12
																	Jadad = NA
Akalin E et al [14]	2003	Case-Control	Kidney and Kidney-pancreas	129	n/a	63/37	13	47	n/a	n/a	100	0	100	450	Ganciclovir PO (3 g/day)	90	12
																	Jadad = NA
Keven K et al [15]	2004	Case-Control	Kidney and Kidney-pancreas	211	49	61/39	24	100	100	0	0	0	0	450	Ganciclovir PO (3 g/day)	90–180	12
																	Jadad = NA
Park J et al [16]	2006	Case-Control	Liver	109	50	53/47	22	0	100	0	100	0	100	450	Ganciclovir PO (3 g/day)	100	12
																	Jadad = NA

## Results

Nine studies met the prospectively defined inclusion criteria: three randomized [7], [9], [10]; three cohort [11]–[13]; and three case-control [14]–[16] studies. All other studies either met the exclusion criteria defined by the absence of a comparator arm (8 studies), or did not meet the inclusion criteria defined by the absence of a valganciclovir arm (44 studies). A total of 1,831 patients were included in our analysis. The characteristics of each study are presented in table 1. Based on their different study designs, we performed several sensitivity analyses and concluded that they can be appropriately combined by the meta-analytic methodology. These analyses are all described below (pages 10 and 13).

### Efficacy Analysis

#### A. Prevention of CMV Disease in All Trials

The efficacy meta-analysis consisted of seven trials comparing valganciclovir against ganciclovir (N = 1,410) [7], [10], [11], [13]–[16]: two randomized [7], [10]; two cohort [11], [13]; and three case-control studies [14]–[16]. Two of the nine identified trials were excluded because comparators other than universal ganciclovir were used in one trial each: one with medium-dose acyclovir [12] and the other with pre-emptive approach [9].

**Figure 2:**
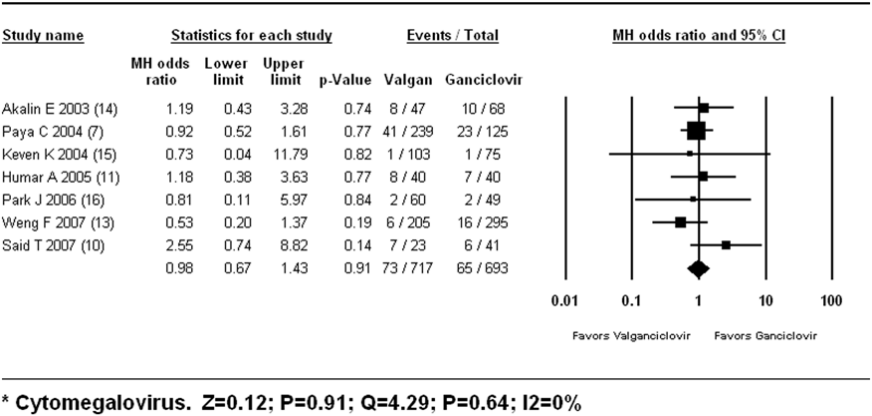
Efficacy: Reduction in CMV Disease.

The overall risk for CMV disease does not differ significantly between the valganciclovir and ganciclovir groups [OR 0.98 (95%CI 0.67 to 1.43; P = 0.92; I^2^ = 0%)] (Fig. 2). The analysis based on the study design shows: All prospective trials [OR 1.11 (95%CI 0.69 to 1.77; P = 0.67; I^2^ = 8%)]; only randomized trials [OR 1.31 (95%CI 0.50 to 3.40; P = 0.58; I^2^ = 54%)]. The analysis by the specific valganciclovir dose shows: valganciclovir 900 mg [OR 1.11 (95%CI 0.69 to 1.77; P = 0.67; I^2^ = 8%)]; valganciclovir 450 mg [OR 0.76 (95%CI 0.40 to 1.44; P = 0.40; I^2^ = 0%)]. The analysis based on allograft type was possible only for kidney/kidney-pancreas recipients [OR 0.99 (0.55 to 1.76); P = 0.97; I^2^ = 26%].

### Sensitivity Analysis

The Weng et al study [13] included patients who did not have documented viral replication but were clinically treated as CMV disease (which could have caused significant confounding results), and the Said et al [10] study gave a regular (90-day) and a shorter (14-day) prophylaxis duration (which could decrease comparability with other studies). These two studies were included/excluded from every efficacy analysis as part of our sensitivity analysis. The overall results with/without these studies (including the 14-day and 90-day arms of Said et al analyzed separately) remained similar. We understand that evaluating a 90-day course against a 14-day course has poor scientific comparability since it is well-known that these regimens may produce different effects. However, since the efficacy results remain similar with both regimens, we kept the 14-day course comparison used for both experimental and control arms. The low-dose valganciclovir (450 mg) efficacy analysis showed an OR 1.08 (95%CI 0.45 to 2.60; P = 0.87; I^2^ = 0%).

In the aim to understand if the overall sample size analyzed for efficacy (Total N = 1,410) would be large enough to detect a potential significant valganciclovir advantage, we calculated the power needed to detect valganciclovir superiority based on a 7% reduction of CMV disease (12% to 5%), as suggested in the original trial by Paya et al. [7]. We found that a total N = 624 would provide a 90% power with a two-tailed alpha of 0.05 to detect a 7% decrease in CMV disease with valganciclovir. Thus, our efficacy meta-analysis sample size (N = 1,410) was adequately powered to detect valganciclovir superiority.

#### B. Prevention of CMV Disease in the D+/R− Subgroup Only

The D+/R− group was separately analyzed for efficacy. The overall efficacy remains similar between valganciclovir and ganciclovir: OR = 0.87 (95%CI 0.52 to 1.45; P = 0.59; I^2^ = 0%). If just the prospective studies are included, the OR is 0.93 (95%CI 0.54 to 1.58; P = 0.78; I^2^ = 0%).

### Safety Analysis

#### A. Risk of Neutropenia

Six studies evaluated the occurrence of neutropenia (ANC<1,500/mm3) during prophylaxis (N = 996) [7], [9], [11], [12], [14], [16]. One study was excluded because it evaluated only leucopenia [15].

**Figure 3:**
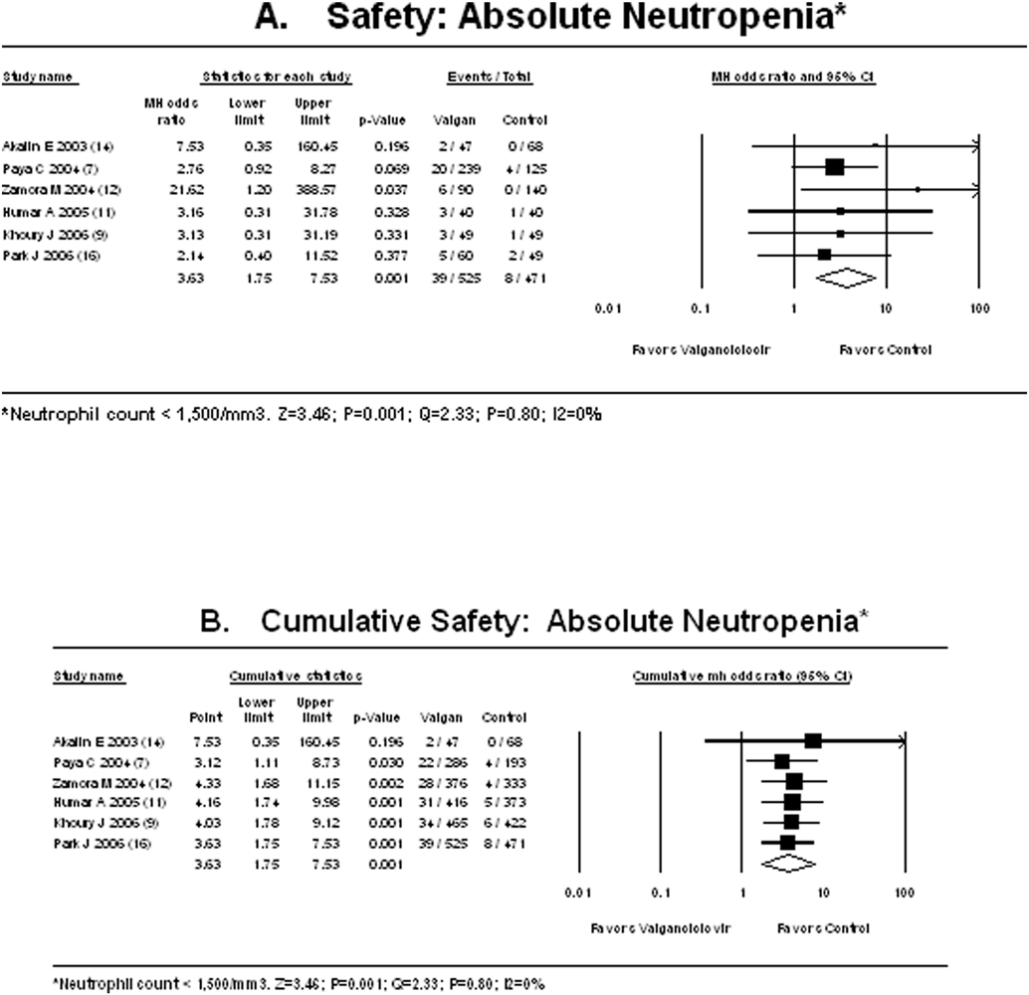
A: Safety: Risk of Absolute Neutropenia. B: Cumulative Safety: Risk of Absolute Neutropenia.

We found that valganciclovir significantly increases the risk of neutropenia by 263% (OR 3.63, 95%CI 1.75 to 7.53; P = 0.001; I^2^ = 0%) compared to all other preventive therapies (Fig. 3A). The cumulative safety meta-analysis by publication year (Fig. 3B) demonstrates that a statistically significant neutropenia with valganciclovir has consistently been observed since the year of 2004. When the studies which compared valganciclovir and ganciclovir only are analyzed, the use of valganciclovir is significantly associated with the risk of neutropenia by 188% (OR 2.88, 95%CI 1.27 to 6.53; P = 0.01; I^2^ = 0%). When the studies which compared valganciclovir with no oral ganciclovir (acyclovir or preemptive) [9], [12] are analyzed, the use of valganciclovir is significantly associated with the risk of neutropenia by 730% (OR 8.30, 95%CI 1.51 to 45.58; P = 0.01; I^2^ = 10%).

The analyses for risk of neutropenia based on the study design shows: All prospective studies [OR 2.87 95%CI 1.15 to 7.14; P = 0.002; I^2^ = 0%]; only prospective studies which used ganciclovir as a comparator [OR 2.83 95%CI 1.05 to 7.62; P = 0.04; I^2^ = 0%]; only randomized studies [OR 2.82 95%CI 1.05 to 7.60; P = 0.04; I^2^ = 0%]). If an ANC cut-off of 1,000 cells/mm^3^ is used, the risk of neutropenia remains significantly elevated (OR 1.97, 95%CI 1.03 to 3.67; P = 0.04; I^2^ = 0%).

### Sensitivity Analysis

Induction therapy with anti-thymocyte preparations and the use of mycophenolate mofetil (MMF) are known potential causes of neutropenia. Two trials did not use MMF [12], [15] but just one reported neutropenia [12], which also showed elevated risk [OR 21.6 95%CI 1.20 to 388.6; P = 0.037]. Valganciclovir is associated with increased risk of neutropenia despite the absence of induction therapy [OR 5.11 95%CI 1.32 to 19.74; P = 0.018; I^2^ = 49%].

Neutropenia may be related to the degree of drug exposure. We performed an analysis according to the valganciclovir dose. The results for the valganciclovir 900 mg/day compared to ganciclovir demonstrate a statistically significantly increased risk of neutropenia (OR 2.87 95%CI 1.15 to 7.14; P = 0.02; I^2^ = 0%), and the results from the valganciclovir 450 mg/day show similarly increased risk, but it was not significantly different from ganciclovir (OR 3.01 95%CI 0.71 to 12.80; P = 0.14; I^2^ = 0%).

For every 24 patients who receive valganciclovir for CMV prophylaxis, one more patient will develop significant neutropenia compared to control treatment strategies across all studies considered (i.e. number needed to harm).

#### B. Opportunistic Bacterial and Fungal Infections

Data were not available for this analysis.

#### C. Survival

Survival could be abstracted from 6 trials [7], [9], [11], [12], [14], [16]. The analysis shows that valganciclovir was not significantly associated with poorer survival (OR 1.29 [95%CI 0.70 to 2.38]; P = 0.40; I^2^ = 0%).

#### D. Risk of Late-Onset CMV Disease in All Trials

Late-onset CMV disease is defined as the occurrence of CMV disease after the completion of universal prophylaxis. The overall risk for late-onset CMV disease with valganciclovir compared to ganciclovir is 1.05 (95%CI 0.67 to 1.64; P = 0.84; I^2^ = 0%). The absence of difference in late-onset CMV-disease is likely secondary to the fact that universal ganciclovir is also associated with late-onset CMV disease [7], [35]–[37]. We also analyzed separately the two trials which did not use ganciclovir prophylaxis as control. The results show a statistically significant 795% higher rate of late-onset CMV disease with valganciclovir compared to control (acyclovir or preemptive therapy) [OR 8.95 (95%CI 1.07 to 74.83; P = 0.04; I^2^ = 0%).

One more patient will develop late-onset CMV disease for every 25 recipients who receive valganciclovir for CMV universal prophylaxis, compared to controls with no oral ganciclovir therapies.

#### E. Risk of Late-Onset CMV Disease in the D+/R− Subgroup Only

Data were not available for this analysis.

#### F. Risk of CMV Disease in Liver Recipients in the Pivotal Trial

The FDA subset analyses [8] showed that the rate of CMV disease was higher in the liver recipients who received valganciclovir (19% [22/118]) than among those who received ganciclovir (12% [7/59]) for prophylaxis. Also, the rates of CMV tissue-invasive disease for liver recipients in the valganciclovir and in the ganciclovir arms were respectively 14% (16/118) and 3% (2/59). Thus, the FDA did not approve valganciclovir for liver recipients. Our multivariate logistic regression analyses based on the above data shows the following: At 6 months, we found that there are statistically significant organ by treatment interactions for both overall CMV disease (p = 0.008) and tissue-invasive CMV (p = 0.009). Specifically, for the liver recipient subgroup, the 6-month risk of having CMV tissue-invasive disease for the valganciclovir (14%) group is 4.5 times [95%CI 1.00 to 20.14] (p = 0.04) the risk in the ganciclovir group (3%). At 12 months, there are also significant organ by treatment interactions for both overall CMV disease (p = 0.07) and tissue-invasive CMV (p = 0.05). The 12-month risk of a liver recipient having tissue-invasive CMV disease for the valganciclovir group (14%) is 3.2 times [95%CI 0.99 to 11.19] (p = 0.05) the risk in the ganciclovir group (5%). The tissue-invasive CMV results indicate a qualitative interaction because the significant result in the liver recipient group was in the opposite direction of the results for other allograft groups.

#### G. Risk of CMV Disease in Liver Recipients in Other Trials

We identified two other published studies which used valganciclovir in liver recipients [16], [38], but were not included in the meta-analysis because both were non-comparative observational studies. Both were retrospective and evaluated universal prophylaxis with valganciclovir. The first [38] described an overall rate of CMV disease (N = 203) of 17%, but the rate in the CMV D+/R− group (N = 59) was 26% with valganciclovir prophylaxis. The second study [16] found an overall (N = 60) rate and CMV D+/R− group (N = 15) rate of 3% and 7%, respectively. Both studies had similar baseline proportion of high-risk patients with CMV D+/R− status [22% [16] and 26% [38]], similar immunosuppressive regimens (FK;MMF;Prednisone) and target FK concentrations, no use of induction, and follow up for 12 or more months. The lack of prospective controls in both studies may account for the differences in the rates of CMV disease. We calculated an exact 95% CI for the CMV disease rates in the high-risk (CMV D+/R−) groups of both trials to compare them with the results from the original trial [7]. We found a rate of 7% (95%CI 0.2% to 32%) for Park et al [16], and 26% (95%CI 15% to 40%) for Jain et al [38]. Both confidence intervals include the estimated CMV disease rate (20%) and CMV tissue-invasive disease (14%) found in the liver recipients from the pivotal trial [7]. Furthermore, there is no significant difference in the CMV disease rates among all three studies (p = 0.2, Chi-square test).

#### H. Publication Bias

**Figure 4:**
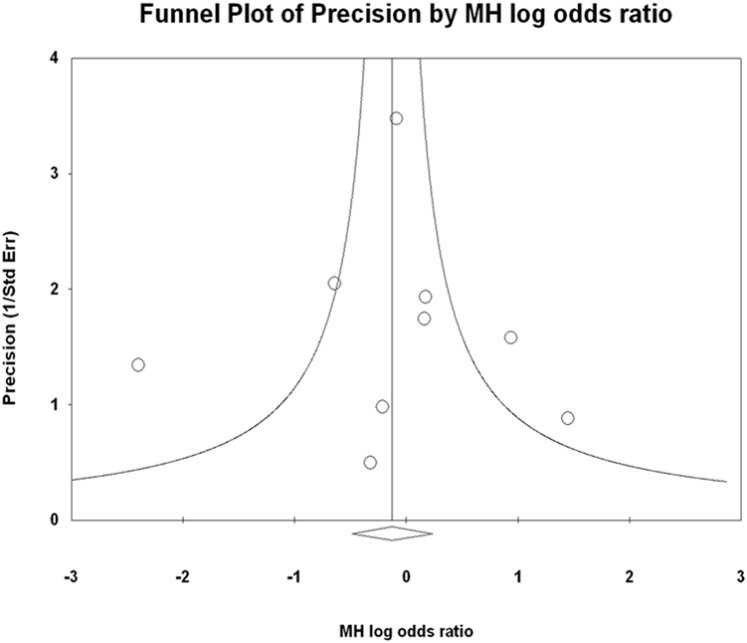
Funnel plot: Evaluation of Publication Bias.

No publication bias was detected by Egger regression (intercept = 0.264; standard Error = 0.777; P = 0.748), or Begg and Mazumdar rank correlation (Kendall's tau = 0.238; P = 0.548). A funnel plot analysis was also performed (figure 4), and despite the small number of studies, no substantial asymmetry was observed.

## Discussion

Our comprehensive evidence-based analysis of published studies exploring CMV prophylactic strategies in SOT patients failed to demonstrate greater efficacy of valganciclovir over standard ganciclovir. We found that universal prophylaxis with valganciclovir is significantly more toxic than with oral ganciclovir (188% higher rate of neutropenia), or non-ganciclovir therapies (730% higher rate of neutropenia), in which both have demonstrated efficacy for CMV prophylaxis [2], [4], [39], [40].

The higher rate of neutropenia observed with valganciclovir is independent of the study design, type of control, or concomitant use of regimes such as T-cell depleting induction therapies or MMF which might also suppress the bone marrow. In addition, the remarkable consistency of the risk estimates for neutropenia seen in our cumulative meta-analysis (Fig. 3B) demonstrates that the risk of neutropenia has been statistically significantly higher with valganciclovir compared to other therapies since 2004. In view of these findings, the question remains as to why valganciclovir is so widely employed by clinicians and surgeons [5], [6] if it is no more effective and less safe than other less costly alternatives?

Despite its low bioavailability, ganciclovir (3 g/day) was found to be an effective suppressant of CMV reactivation and clinical disease in SOT patients who took it for 3 months prophylactically after transplant [2], [4], [35], [41], [42]. Valganciclovir is the L-valyl ester prodrug of ganciclovir that has greater oral bioavailability and yields plasma levels of ganciclovir that are comparable to, or even higher than, intravenous administration of 5 mg/kg ganciclovir [43], [44]. Although both drugs preferentially inhibit viral DNA polymerases, they also interact with host enzymes, resulting in varying degrees of marrow suppression. It stands to reason, therefore, that the more highly available valganciclovir would tend to have greater marrow toxicity as a consequence of its higher and more prolonged plasma concentrations of the parent drug, ganciclovir. Indeed, the pivotal study [7] comparing the two agents revealed 8.2% and 3.2% incidences of neutropenia, for valganciclovir and ganciclovir, respectively. Although one might expect greater efficacy to accompany the higher plasma levels of ganciclovir that are seen with oral valganciclovir, this has not proved to be the case by our comprehensive analysis. This intuitive clinical reasoning commonly used for the treatment approach may not be applicable for the prophylactic approach, in which higher drug concentrations may not translate into further benefits, but rather more side effects.

The rates of neutropenia that we found most likely represent an underestimation of this important clinical problem, due to the variability and imprecision in reporting the degree of neutropenia in the various studies. ANC<1,000 and 1,500 mm^3^ were considered representative of neutropenia in most studies, but the depth of neutropenia was not specifically or systematically reported. Thus, it is not known what proportion of patients had more severe levels of neutropenia, of which could impart a substantially greater infection risk to the patient. Surrogate end-points for safety commonly used in prophylaxis studies (i.e. ‘stopping valganciclovir’, ‘requiring growth factor’) are influenced by subjective clinical thresholds which differ from study-to-study and from hospital-to-hospital; may not correlate with the magnitude of neutropenia or actual risk of developing infections; and may be potentially misleading about the actual valganciclovir effect in SOT patients [45], [46]. The risk of serious opportunistic infections secondary to valganciclovir-induced neutropenia could not be analyzed, given the lack of data, but it is notable that there was a trend toward increased risk of death in the population who received valganciclovir. However, a direct cause-effect between valganciclovir and higher risk of infectious diseases is yet to be demonstrated in future studies, in which underreporting could be avoided by the systematic collection of all post-transplant infectious diseases.

Another potential limitation of our results is related to the lack of trial reporting on the rate of CMV resistance to ganciclovir in most studies. However, the randomized prospective study by Boivin et al [47] demonstrated a very low incidence of CMV genotypic resistance (1.9%) in multiple transplant centers. Additionally, the genotypic presence of mutations is not necessarily associated with clinical resistance. Other limitations that are intrinsic to the statistical methodology used in our study include: 1) the use of aggregated data rather than individual data; and 2) the normality assumption, which may not hold for subgroup analysis. Of note, the Jadad scores used to evaluate the quality of included studies have been validated only in randomized trials, so the scores of the non-randomized studies included in our analyses may not have the same reliability.

Paya et al [7] reported that valganciclovir was “as clinically effective and well-tolerated as oral ganciclovir for CMV prevention in high risk SOT recipients”. According to the CONSORT statement [31], [48], “…equivalence trials aim to determine whether intervention is therapeutically similar to another, usually an existing treatment…and non-inferiority trials seek to determine a new treatment is no worse than a reference treatment”. Therefore, based on the fact this trial [7] had a non-inferiority design, the correct interpretation would be that valganciclovir is ‘no worse than’ or ‘not inferior to’ ganciclovir. A non-inferiority margin of −5% or −Δ = −5.0% (i.e. lower bound of 95% CI) was used in the valganciclovir trial [7], which means that valganciclovir could show up to a 5% higher rate (absolute difference) of CMV disease than ganciclovir and still be deemed non-inferior to ganciclovir. The results of the intent-to-treat (ITT) analysis showed a −Δ = −4.2% (3.1% [95%CI −4.2% to 11%]) at 6 months; and a −Δ = −6.8% (1.5% (95%CI −6.8% to 9.8%]) at 12 months; for the Per Protocol (PP) at 6 months, the −Δ = 5.1% (3.9% (95%CI −5.1% to 12.9%]). In principle, the use of the ITT population in a non-inferiority designed trial can artificially enhance the claim of non-inferiority by diluting the real treatment differences [49]–[51], i.e. falsely accepting a new truly inferior treatment as non-inferior (type I error). Hence, if the primary outcome of this study was based on the differences in CMV disease rates in the PP population analysis at 6 months (−Δ = −5.1%), or on the ITT population analysis at 12 months (−Δ = −6.8%), valganciclovir would not be considered non-inferior to ganciclovir.

Based on the original trial [7], valganciclovir appears to be non-inferior to ganciclovir; however, the fact that both 6-month per protocol and 12-month intention-to-treat analyses failed to meet the predefined non-inferiority criteria is troublesome. We calculated that a sample size of 624 patients would have been adequate to detect valganciclovir superiority in this trial. It may be argued that the pivotal trial sample was not large enough to achieve this end; however, our data, comprising a cohort of 1,410 patients, did not even show a trend for better efficacy based on the point estimate (OR = 0.98) or statistical significance (p = 0.91). Thus, even at sufficient numbers of patients for evaluation, there is no suggestion of valganciclovir superiority. Our results were not changed by the analyses of different study designs or D+/R− serostatus subgroup. The inclusion of several non-randomized trials would tend to overestimate the efficacy effect [52], [53], or give similar results [54], [55], compared with randomized trials. Our inclusion of these non-randomized trials might have favored a valganciclovir beneficial effect, but in fact, the results still showed no such superiority.

The high rate of tissue-invasive CMV disease observed in the liver recipients of the pivotal trial [7] remains an important safety concern. The statistically significant treatment by allograft interaction both at 6 and at 12 months analyses suggest that valganciclovir may be harmful to liver recipients. Similar high rates of tissue-invasive CMV disease have also been observed in a large retrospective study [38] of liver transplant recipients done thereafter. Nonetheless, 61% [6] of liver transplant programs now use valganciclovir prophylaxis despite the potentially negative effects (tissue-invasive CMV disease) of this drug in this patient population.

Singh et al [56] have argued that complete suppression of CMV replication as a consequence of universal valganciclovir prophylaxis will likely not permit the host immune system to be exposed to naturally occurring low-level episodes of CMV antigenemia. Accordingly, the host remains “naïve” to CMV antigens until valganciclovir prophylaxis ceases, at which time CMV reactivation may occur without benefit of primed host responses. These so-called “late-onset CMV infections” would therefore be expected to occur more often with an anti-CMV agent that strongly suppress replication, such as valganciclovir, as compared with acyclovir or ganciclovir. In fact, we observed an 800% significant higher risk of late-onset CMV disease with valganciclovir compared to acyclovir prophylaxis or preemptive therapy, both of which may allow for some intermittent replication of CMV and therefore, host immunologic exposure. Our results indicate that one more patient will develop late-onset CMV disease for every 25 recipients who receive valganciclovir for CMV prophylaxis. Considering that late-onset CMV disease is by itself a significant risk factor for death in transplant recipients [57], this complication has important clinical consequences.

Low-dose valganciclovir (450 mg daily) is an attractive potential alternative to the currently recommended dose of 900 mg daily, because it would presumably be associated with less toxicity, and could allow for some low-level CMV replication that might be immunologically advantageous. In fact, our analysis did not show a significantly higher rate of neutropenia with valganciclovir 450 mg daily compared with ganciclovir, but the findings suggested a 200% increase in the risk of neutropenia. Since the sample sizes were limited, all controls used ganciclovir (which also causes neutropenia), and all low-dose studies were retrospective, the estimated risk of neutropenia associated with the 450 mg valganciclovir dose may be understated here. While a prospective study evaluating the efficacy and safety of the 450 mg dose valganciclovir could add important information, the current data does not suggest that this dose would cause less neutropenia than the 900 mg dose.

A pre-emptive approach to prevention of CMV disease in the SOT patient is also effective, and may lead to less drug exposure than universal prophylaxis. However, once a positive CMV test develops and triggers antiviral treatment, the typical duration of therapy and maintenance is approximately 4–24 weeks [6], which is of a similar duration to universal prophylaxis. Therefore, one would expect that the side-effect profile of valganciclovir during pre-emptive treatment would be comparable to that seen with universal prophylaxis. The reported rate of leucopenia (12–82%) in observational trials with pre-emptive approach [17]–[24] is similar or higher than the rates seen with valganciclovir universal prophylaxis. On the other hand, ganciclovir is associated with significantly lower risk of neutropenia in our study, and has consistently demonstrated high efficacy when used preemptively [2]–[4]. Compared to valganciclovir and IV ganciclovir, oral ganciclovir has also demonstrated efficacy for preemptive prevention of CMV disease [18], [58]. Therefore, based on available safety and efficacy data, oral ganciclovir should be considered the most reasonable choice for the pre-emptive approach following SOT.

Approximately 25,000 solid-organ transplants are performed every year in the US, and according to a recent survey, valganciclovir prophylaxis is being given to 62% of D+/R−, 53% of D+/R+, 48% of D−/R+, and 11% of D−/R− patients [5]. Thus, we estimate that 12,000 to 13,000 recipients are prescribed valganciclovir prophylaxis every year. Based on the approximately 8% neutropenia rate observed in the pivotal trial [7] and in our study, 1,000 patients would develop significant neutropenia related to valganciclovir each year. These numbers are likely an underestimation because currently, many more patients are taking valganciclovir prophylaxis for off-label indications: T-cell depleting induction therapies; allograft rejections; prophylaxis for D+/R+, D−/R+, and D−/R− recipients; CMV viremia without symptoms; and preemptive therapy. In fact, the most recent survey showed that 61% of the transplant centers are using valganciclovir for CMV prophylaxis in liver recipients [6], despite the absence of an FDA indication. Given the adverse effect profile noted in our analysis, the lack of superiority of the drug against other standard therapies, and the increase in CMV tissue-invasive disease, valganciclovir prophylaxis should be contraindicated for all liver recipients until further data documents its safety in this patient population. In fact, valganciclovir has not been the prophylaxis regimen of choice for the high-risk liver transplant recipients in our center since September 2003.

In conclusion, based on its significantly higher risk of neutropenia, late-onset CMV disease, and its lack of superiority against other standard therapies, valganciclovir is not the preferred option as a first-line agent for CMV preemptive or universal prophylaxis in SOT recipients, especially given the availability of other efficacious, safer, and lower cost oral alternatives (e.g. high-dose acyclovir, valacyclovir, ganciclovir).

## Supporting Information

Table S1 (0.06 MB DOC)
