# Supplementary material for: Valganciclovir for Cytomegalovirus Prevention in Solid Organ Transplant Patients: An Evidence-Based Reassessment of Safety and Efficacy
Source: PLoS One. 2009 May 13;4(5):e5512. doi: 10.1371/journal.pone.0005512 (PMC2677673; doi:10.1371/journal.pone.0005512)
Supplement: Table S1 — (0.06 MB DOC) [file pone.0005512.s001.doc]

**MOOSE Checklist**

**Valganciclovir for Cytomegalovirus Prevention:**

**An Evidence-Based Reassessment of Safety and Efficacy.**

Corresponding Author:

Andre Kalil, M.D.

Associate Professor of Medicine, University of Nebraska

Associate Director, Immunocompromised Host ID Program

985400 Nebraska Medical Center

Omaha, NE 68198-5400

E-mail: akalil@unmc.edu

| **Criteria** | | **Brief description of how the criteria were handled in the meta-analysis** |
| --- | --- | --- |
| **Reporting of background should include** | |  |
| √ | Problem definition | Cytomegalovirus is the most common opportunistic infection in solid organ transplant patients. Valganciclovir, an anti-cytomegalovirus drug, is the most used prophylaxis regimen for this infection today. However, solid evidence for its safety and efficacy is still lacking. The most prominent side effect of this drug is neutropenia, which can further predispose the transplant patient to serious infections. |
| √ | Hypothesis statement | Valganciclovir is superior and safer than other standard therapies |
| √ | Description of study outcomes | Cytomegalovirus Disease. |
| √ | Type of exposure or intervention used | Valganciclovir. |
| √ | Type of study designs used | We included randomized, prospective cohort studies, and case-control studies. We excluded single-arm observational studies. |
| √ | Study population | Recipients of all solid organ transplants. |
| **Reporting of search strategy should include** | |  |
| √ | Qualifications of searchers | The credentials all investigators are indicated in the author list. |
| √ | Search strategy, including time period included in the synthesis and keywords | PubMed, Embase, and Cochrane Library from inception to May 2008. All keywords are described on page 6. |
| √ | Databases and registries searched | PubMed, EMBASE, and Cochrane databases. |
| √ | Search software used, name and version, including special features | RefWorks was used to merge all abstracted citations and to eliminate duplications. A specific search software was not used. |
| √ | Use of hand searching | Hand-searched bibliographies were performed for scientific meetings databases. |
| √ | List of citations located and those excluded, including justifications | The literature search process is outlined in the trial flow chart. The entire citation list is available upon request. |
| √ | Method of addressing articles published in languages other than English | We placed no restrictions on language. No translation was necessary. |
| √ | Method of handling abstracts and unpublished studies | No abstracts of unpublished studies were found for the study. |
| √ | Description of any contact with authors | We contacted authors to clarify some of the study reports, but no new analyses were performed. |
| **Reporting of methods should include** | |  |
| √ | Description of relevance or appropriateness of studies assembled for assessing the hypothesis to be tested | The inclusion and exclusion criteria were individually explained in the methods section. |
| √ | Rationale for the selection and coding of data | The variables and data selected for this study were pertinent to this specific patient population. |
| √ | Assessment of confounding | Several sensitivity analyses addressing possible confounding factors were performed and described in the results section. |
| √ | Assessment of study quality, including blinding of quality assessors; stratification or regression on possible predictors of study results | Stratification analyses were performed for the type of study design, type of allograft, immunosuppressive regimen, valganciclovir dosing, and degrees of neutropenia. |
| √ | Assessment of heterogeneity | Both Cochrane’s Q test of heterogeneity and I^2^ statistic were used for within and between-study heterogeneity. |
| √ | Description of statistical methods in sufficient detail to be replicated | We comprehensively described all statistical methods used for this meta-analysis in the methods section. |
| √ | Provision of appropriate tables and graphics | This manuscript has one table describing the characteristics of each included study, one figure with the trial flow, and three figures with the forest plots. |
| **Reporting of results should include** | |  |
| √ | Graph summarizing individual study estimates and overall estimate | These graphs can be seen in figures 2, 3A, and 3B. |
| √ | Table giving descriptive information for each study included | Table 1. |
| √ | Results of sensitivity testing | Results section on pages 10 and 13. |
| √ | Indication of statistical uncertainty of findings | The uncertainty of findings was evaluated by 95% confidence intervals, which were presented with all summary estimates, and the uncertainty of homogeneity was evaluated by I^2^ statistics, also presented with all estimates. |
| **Reporting of discussion should include** | |  |
| √ | Quantitative assessment of bias | Sensitivity analyses indicate heterogeneity in strengths of the association due to most common biases in observational studies. |
| √ | Justification for exclusion | We excluded studies that had not adjusted for or were standardized by age, a potential confounder, and used different exposure or outcome assessment for the comparison groups. Those studies that were excluded also indicate positive association, as noted in the discussion. |
| √ | Assessment of quality of included studies | We discussed the results of the sensitivity analyses, and potential reasons for the observed heterogeneity. |
| **Reporting of conclusions should include** | |  |
| √ | Consideration of alternative explanations for observed results | We explicated in depth the several alternatives explanations in the discussion section on pages 18-23. |
| √ | Generalization of the conclusions | This is one of the main strengths of our study, since we included several studies which evaluated the real life experience (post phase III trial) with this drug. |
| √ | Guidelines for future research | Unless the safety and efficacy of valganciclovir is further evaluated in larger randomized studies, transplant physicians should used more caution before using this drug for prophylaxis. |
| √ | Disclosure of funding source | No funding was received in support of this study. |
